# Potential role of p53 on metallothionein induction in human epithelial breast cancer cells

**DOI:** 10.1038/sj.bjc.6600549

**Published:** 2002-10-21

**Authors:** L Z Fan, M G Cherian

**Affiliations:** Department of Pathology, University of Western Ontario, London, Ontario, N6A 5C1, Canada

**Keywords:** metallothionein, p53, apoptosis, breast cancer

## Abstract

The expression and induction of metallothionein has been associated with protection against oxidative stress and apoptosis. This study examines the effect of tumour suppressor protein p53 on metallothionein expression following CdCl_2_ treatment in eight human epithelial breast cancer cell lines differing in p53 and oestrogen-receptor status. Cells were treated with 10 μM CdCl_2_ for 24 h and metallothionein protein levels were measured by cadmium binding assay. MCF7 cells which are p53-positive (p53+) and oestrogen-receptor-positive showed a large induction in metallothionein synthesis by 10.79±1.36-fold. Other breast cancer cell lines which are p53-negative (p53−) and oestrogen-receptor-negative or weakly oestrogen-receptor-positive showed a small induction ranging from 1.40±0.10 to 3.65±0.30-fold. RT–PCR analysis showed an induction of metallothionein mRNA in MCF7 cells by about 1.61±0.08-fold, while in HCC1806 cells (p53−, oestrogen-receptor-negative) by 1.11±0.13-fold, and in MDA-MB-231 (p53−, oestrogen-receptor-negative) by 1.25±0.06-fold. Metallothionein localisation was determined by immunohistochemical staining. Prior to metal treatment, metallothionein was localised mainly in the cytoplasm of MCF7 and MDA-MB-231 cells. After treatment with 10 μM CdCl_2_ for 24 h, MCF7 cells showed intense nuclear and cytoplasmic staining for metallothionein, while MDA-MB-231 cells showed staining in the cytoplasm with weak nuclear staining. Apoptosis induced by 10–40 μM CdCl_2_ at time points between 4 and 48 h was examined with TUNEL assay. In MCF7 cells, apoptosis increased with higher concentrations of CdCl_2_, it peaked at 6–8 h and appeared again at 48 h for all concentrations of CdCl_2_ tested. In MDA-MB-231 cells, apoptosis remained at low levels for 10–40 μM CdCl_2_ at all time points. Studies on cadmium uptake showed similar uptake and accumulation of cadmium at 8 and 24 h in all the cell lines. The data demonstrate that treatment of epithelial breast cancer cells with 10 μM CdCl_2_ for 24 h caused a greater induction of metallothionein protein and mRNA expression in p53+ and oestrogen-receptor-positive cells as compared to p53− and oestrogen-receptor-negative or weakly oestrogen-receptor-positive cells. This effect may be associated with the occurrence of apoptosis and suggests a role for p53 and oestrogen-receptor on the expression and induction of metallothionein in epithelial cells.

*British Journal of Cancer* (2002) **87**, 1019–1026. doi:10.1038/sj.bjc.6600549 www.bjcancer.com

© 2002 Cancer Research UK

## 

The expression and induction of metallothionein (MT) has generally been associated with protection against oxidative stress and apoptosis. It has been reported that MT-null mice and cells derived from them were more sensitive to the toxic effects of heavy metals such as cadmium (Klaassen *et al*, 1999), toxic agents such as hydrogen peroxide and tert-butyl hydroperoxide (Chubatsu and Meneghini, 1993; Schwarz *et al*, 1994), and radiation exposure (Cai *et al*, 1999) as compared to MT-wildtype and MT I-overexpressing mice. Cells pre-induced to express MT were also more resistant against the toxic effects of cadmium (Goering and Klaassen, 1984; Klaassen *et al*, 1999) and radiation exposure (Cai *et al*, 1999) as compared to control cells. In human T cell and breast carcinoma cell lines, down-regulation of MT with antisense MT oligomers not only inhibited growth of the cells, but also activated apoptosis (Abedel-Mageed and Agrawal, 1997; Tsangaris and Tzortzatou-Stathopoulou, 1998). In studies using tumour cell lines, it was found that tumour cells with a high expression of MT were more resistant against the toxic effects of anticancer agents such as cisplatin as well as radiation exposure (Kondo *et al*, 1995; Lazo *et al*, 1998). In addition, in both human primary hepatocellular carcinoma and metastatic carcinoma, the MT levels were low and had higher number of apoptotic cells as compared to normal liver (Cai *et al*, 1998; Deng *et al*, 1998).

The potential role of MT on modulation of apoptosis is unclear. Antioxidant properties of MT may contribute to its protective effects (Greenstock *et al*, 1987; Cai *et al*, 1996). In fact, the synthesis of MT was shown to be induced several-fold during oxidative stress (Sato and Bremner, 1993) to protect the cells against cytotoxicity (Aschner *et al*, 1998) and DNA damage (Cai *et al*, 1995). In particular, MT synthesis is induced following treatment with cadmium, an environmental pollutant that is able to cause oxidative stress, DNA damage and apoptosis (El Azzouzi *et al*, 1994; Yang *et al*, 1997). The protective role of MT may also depend on the nuclear/cytoplasmic localisation of MT in the cell. In general, cytoplasmic MT protects against cytotoxicity whereas nuclear MT protects against genotoxicity (Schwarz *et al*, 1994; Woo and Lazo, 1997; Cherian and Apostolova, 2000).

The tumour suppressor protein p53 is one of the most frequently activated proteins in apoptosis. p53 is able to respond to different cellular stresses such as DNA damage, hypoxia, oxidative stress, and oncogene activation. Upon activation, p53 initiates a number of cellular activities that can ultimately culminate in G1 or G2 cell cycle arrest and DNA repair, apoptosis, or other cellular changes (Schwartz and Rotter, 1998). As a critical cellular mediator of the response to genotoxic damage, P53 has a direct role in maintaining the integrity of the genome. Loss of p53 activity has been associated with tumour progression and unfavourable prognosis of the tumour (Symonds *et al*, 1994). Recently, it was found that an association exists between MT expression and p53 expression in small cell carcinoma of the lung (Joseph *et al*, 2001). Since both MT and p53 are involved in responding to oxidative stress and apoptosis, the present study was undertaken to investigate the effect of p53 on MT expression and induction in human epithelial breast cancer cells.

MT expression in breast cancer has been associated with a lower number of apoptotic cells (Abdel-Mageed and Agrawal, 1997) and a poor prognosis of the cancer (Haerslev *et al*, 1995; Oyama *et al*, 1996). Recently, certain studies have demonstrated that oestrogen receptor-positive (ER+) breast cancer cells showed lower expression of MT as compared to oestrogen receptor-negative (ER−) cells (Friedline *et al*, 1998). Possibly, p53 may be another factor in determining MT expression. Since p53 is mutated in more than 70% of human breast cancers (Callahan and Campbell, 1989), it is possible to examine the effects of p53-positive (p53+) as well as p53-negative (p53−) breast cancer cell lines. These cell lines also show differential expression of oestrogen receptor. In this study, MT expression and induction following cadmium exposure were investigated in eight epithelial breast cancer cell lines differing in p53 and ER expression.

## MATERIALS AND METHODS

### Cell culture

Human epithelial breast cancer cell lines MCF7 and HCC1806 were obtained from Dr Rodenheiser; HCC1419 and HCC1569 from ATCC; MDA-MB-231, MDA-MB-468 and HS578t from Drs Laird and Lala; and MDA-MB-435 from Drs Ferguson and Koropatnick. MCF7 cells were grown in Dulbecco's Modified Essential Medium (DMEM) supplemented with 10% foetal bovine serum (FBS). HCC1806, HCC1419, HCC1569, MDA-MB-231 and MDA-MB-468 cells were grown in RPMI1640 medium supplemented with 10% FBS. HS578t cells were grown in RPMI1640 medium supplemented with 10% FBS and 10 μg ml^−1^ of insulin. MDA-MB-435 cells were grown in Minimum Essential Medium Alpha containing ribonucleosides and deoxyribonucleosides and supplemented with 10% FBS. The epithelial breast cancer cell lines differ in p53 and ER expression (Table 1

**Table 1:**
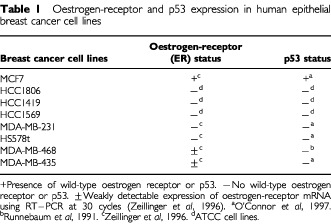
Oestrogen-receptor and p53 expression in human epithelial breast cancer cell lines

).

### Cell viability assay

Cell viability following CdCl_2_ treatment was measured using Intergen's Procheck Cell Viability Assay (Intergen Co., Purchase, NY, U.S.A.) based on the conversion of XTT (sodium3,3′-{1-[(phenylamino) carbonyl]-3,4 - tetrazolium}bis(4-methoxy-6-nitro)benzene sulphonic acid) from an oxidized tetrazole to a reduced formazan (Scudiero *et al*, 1988). Cells were seeded into 96-well plates with 10^4^ cells well^−1^ and allowed to attach for 24 h. Cells were treated with 10–100 μM of CdCl_2_ for 12 h and then washed twice with PBS to stop the treatment. To the wells, 100 μl of media and 20 μl of the assay reagent were added and cells were incubated under growth conditions for 4 h. Optical densities were read at 475 nm in a microplate spectrophotometer and cell viability was expressed as a per cent of control.

### Estimation of MT

Cells grown in 75-cm^2^ T flasks were treated with 10 μM CdCl_2_ for 24 h and then washed twice with PBS. Cells were collected by trypsinisation followed by centrifugation at 2000 r.p.m. for 2 min. Quantification of MT was performed by a ^109^cadmium-haeme saturation assay as previously described (Eaton and Cherian, 1991). Briefly, the cell pellet was resuspended in 630 μl of deionised water and frozen and thawed to lyse the cells. To a 300 μl aliquot, 1 ml of a 30 mM Tris-HCl buffer (pH 8.0) and 1 ml of a 5 p.p.m. ^109^Cd solution with known specific activity were added to saturate the metal-binding sites of MT. Rat haemolysate was added to remove excess Cd, followed by heat treatment in a water bath to precipitate Cd-hemoglobin and other proteins, with the exception of MT which is heat stable. The denatured proteins were then removed by centrifugation at 10 000 r.p.m. for 2 min. The steps of haemolysate addition, heat denaturation and centrifugation were repeated three times. The Cd concentrations in the final supernatant were calculated from the radioactivity of the ^109^Cd measured by a γ counter (1272 Clinigamma, LKB Wallac; Turku, Finland), with a counting efficiency of 75%, and were converted to MT concentration on the basis of 7 g atoms of cadmium/MT. An additional aliquot of the lysed cell suspension was assayed for protein with Bio-Rad Protein Assay (Bio-Rad Laboratories, Hercules, CA, USA). The total MT concentrations in the cells were expressed as μg MT mg protein^−1^.

### Immunohistochemical staining for MT

Cells were seeded in 4-chamber slides with 5×10^4^ cells per chamber and allowed to attach for 24 h. Cells were then treated with 10 μM CdCl_2_ for 24 h and then washed twice with PBS. The immunohistochemical staining technique for MT was previously described (Deng *et al*, 1998). Briefly, cells were fixed in 1% paraformaldehyde in PBS for 10 min at room temperature. Endogenous peroxidase activity was inactivated by quenching the cells in 3.0% hydrogen peroxide for 5 min. Cells were incubated with 10% normal goat serum for 45 min, followed by incubation with polyclonal rabbit anti-MT serum (1 : 400) for 2 h at room temperature. This antibody was generated against a polymer of rat liver MT but readily cross-reacts with human MT. Normal rabbit serum substituted for the primary antibody was used as negative control. Subsequently, cells were incubated with biotinylated goat anti-rabbit IgG and then with avidin-biotin horseradish peroxidase complex following the manufacturer's instruction (ABC Kit, Vector Laboratories, Burlingame, CA, USA). Staining was developed with 0.05% 3,3′-diaminobenzidine tetrahydrochloride (DAB) with 0.33% hydrogen peroxide, counterstained with haematoxylin and 0.3% ammonia solution, dehydrated and mounted.

### RNA isolation and RT–PCR

Cells grown in 75-cm^2^ T flasks were treated with 10 μM CdCl_2_ for 24 h and then washed twice with PBS. Total RNA was isolated from the cells with TRIzol Reagent (GIBCO BRL, Life Technologies, Grand Island, NY, USA) according to the manufacturer's instructions. RNA was extracted with chloroform followed by centrifugation to separate the solution into aqueous and organic phases. RNA was recovered from the aqueous phase by precipitation with isopropyl alcohol and suspended in diethyl pyrocarbonate-treated water. The concentration of isolated RNA was measured by spectrophotometry at 260 nm and RNA samples with purity greater than 1.6 (260/280 nm ratio) were used for reverse transcription.

First strand cDNA synthesis was performed using Superscipt-II system (GIBCO BRL, Life Technologies, Gaithersburg, MD, USA). RNA was added to Oligo (dT) primers (GIBCO BRL), denatured at 70°C, and quenched on ice for 10 min. Five micrograms of RNA were reverse transcribed for 50 min at 42°C with superscript Moloney murine leukaemia virus reverse transcriptase (GIBCO BRL) and dNTP in a total reaction volume of 20 μl. The reaction was terminated by a 7 min incubation at 70°C.

The resulting reverse transcribed product was then used for PCR amplification performed with the oligonucleotide primers specific for human MT-II (GIBCO BRL). MT-II primer sequences were as follows: 5-CTC TTC AGC ACG CCA TGG AT-3 (sense) and 5-CGC GTT CTT TAC ATC TGG GA-3 (antisense). The predicted size of the amplified product (cDNA) was 203 bp for MT-II. PCR was performed under the following conditions with a thermal cycler. Each sample contained 1 μM of the sense and antisense primers for MT-II, 1×PCR buffer (GIBCO BRL), 1.0 mM MgCl_2_ (GIBCO BRL), 250 μM dNTP (GIBCO BRL) and 2.5 U μl^−1^ *Taq* DNA polymerase (GIBCO BRL) in a final volume of 50 μl. PCR was carried out for 23 cycles with denaturation at 94°C for 45 s, annealing at 55°C for 30 s, and extension at 72°C for 1 min 30 s, with a final elongation step at 72°C for 10 min. Simultaneously, a housekeeping gene, β-actin, was amplified in a separate set of tubes using the same RT product and similar cycling parameters. β-actin primer sequences were as follows: 5′-CCT CTA TTC CAA CAC AGT GC-3′ (sense) and 5′-CAT CGT ACT CCT GCT TGC TG-3′ (antisense), with predicted product size of 210 bp.

Following PCR, a 10 μl aliquot of the RT–PCR cDNA was electrophoresed in 3% agarose gel in Tris/acetic acid/EDTA (TAE) buffer for 70 min at 100 V, stained with ethidium bromide, and visualised with UV light. The intensity of the cDNA bands was analysed by densitometry, and MT-II mRNA expression was normalised with β-actin.

### *In situ* apoptosis detection

Cells were seeded in 8-chamber slides with 5×10^4^ cells per chamber and allowed to attach for 24 h. Cells were then treated with CdCl_2_ for varying time points and treatment was stopped by washing twice with PBS. The method of TdT-mediated deoxyribonucleotide triphosphate-digoxigen nick end labelling (TUNEL) was used for *in situ* labelling of apoptotic cells. Staining was conducted according to the manufacturer's instruction (ApopTag Peroxidase *In Situ* Apoptosis Detection Kit, Intergen Co.). Briefly, cells were fixed in 1% paraformaldehyde in PBS for 10 min at room temperature, followed by post-fix in pre-cooled ethanol : acetic acid 2 : 1 for 5 min at −20°C. Endogenous peroxidase was inactivated by quenching the cells in 3.0% hydrogen peroxide for 5 min. Terminal deoxynucleotidyl transferase (TdT) enzyme and digoxigenin-labelled dUTP were applied to the sections for 1 h at 37°C. Cells were then washed in stop/wash buffer and treated with anti-digoxigenin peroxidase conjugate for 30 min at room temperature. Staining was developed with 0.05% 3,3′-diaminobenzidine tetrahydrochloride (DAB) with 0.33% hydrogen peroxide, counterstained with haematoxylin and 0.3% ammonia solution, dehydrated and mounted. Apoptotic cells were examined at 400× magnification, counted in 10 randomly selected fields, and expressed as a per cent of total cells.

### Determination of CdCl_2_ uptake

Cells grown in 75-cm^2^ T flasks were treated with 10 μM of ^109^CdCl_2_ and incubated as described earlier for 8 or 24 h, and then washed twice with PBS. Cells were collected by trypsinisation followed by centrifugation at 2000 r.p.m. for 2 min. The cell pellet was resuspended in deionised water and frozen and thawed to lyse the cells. Disintegrations per minute (d.p.m.) of ^109^CdCl_2_ taken up by the cells were measured by a γ counter (1272 Clinigamma, LKB Wallac) with a counting efficiency of 75%. An additional aliquot of the lysed cell suspension was assayed for protein with Bio-Rad Protein Assay (Bio-Rad Laboratories) and d.p.m. was expressed as d.p.m. mg protein^−1^.

### Statistical analysis

Results are expressed as mean±s.e. The statistical evaluation of the results was performed by one-way ANOVA analysis, followed by student's *t*-test using the error calculated from ANOVA. Significance was established at *P*<0.05.

## RESULTS

### Cell viability after exposure to cadmium

**Figure 1:**
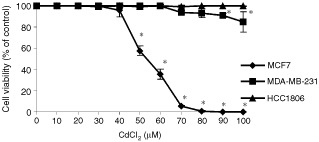
Effects of CdCl_2_ on cell viability. MCF7, MDA-MB-231, and HCC1806 cells were treated with various concentrations of CdCl_2_ for 12 h. Cell viability was determined with Procheck Cell Viability Assay and expressed as a per cent of control. Results are mean±s.e. of three independent experiments. * Significantly different from control at *P*<0.05.

The cell viability of cell lines MCF7 (ER+, p53+), HCC1806 (ER−, p53−) and MDA-MB-231 (ER−, p53−) was assessed after exposure to 10–100 μM CdCl_2_ for 12 h (Figure 1

). The viability of MCF7 cells was unaffected by 10–30 μM CdCl_2_ treatment for 12 h, but decreased to 96% in response to 40 μM CdCl_2_. The viability of MCF7 cells further decreased as the concentration of CdCl_2_ was increased, and was 0% with 80 μM CdCl_2_ exposure. Both HCC1806 and MDA-MB-231 cells were more resistant to CdCl_2_ treatment. HCC1806 cells did not show any decrease in cell viability in response to 10–100 μM CdCl_2_. MDA-MB-231 cells showed a 6% decrease in viability after exposure to 70 μM CdCl_2_, and at 100 μM CdCl_2_ the viability was 85%.

### Induction of MT by cadmium

**Figure 2:**
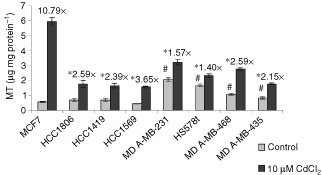
Induction of MT expression with CdCl_2_. Epithelial breast cancer cells were treated with 10 μM CdCl_2_ for 24 h. MT protein levels were determined with ^109^Cadmium-haeme assay and expressed as μg MT mg total protein^−1^. Results are mean±s.e. of three independent experiments. *Ratio of induced MT level/basal MT level is significantly different from MCF7 cells at *P*<0.05. ^#^ Basal MT level is significantly different from MCF7 cells at *P*<0.05.

When these cells were treated to ZnSO_4_ (30–60 μM), no significant differences in MT induction were observed in the different cell lines (data not shown). When cells were treated with oestrogen (1–100 nM), there was no induction of MT (data not shown). The induction of MT by cadmium was examined in eight epithelial breast cancer cell lines differing in p53 and ER status (Table 1). Cells were treated with 10 μM of CdCl_2_ for 24 h to induce MT expression and basal and induced MT levels were measured with the ^109^Cd-haeme assay (Figure 2

). The cell lines showed a range of basal MT levels, with MCF7 at 0.55±0.05 μg MT mg protein^−1^ and the other cell lines at 0.43±0.02 to 2.06±0.11 μg MT mg protein^−1^. Following the induction of MT with 10 μM CdCl_2_ for 24 h, MCF7 cells showed the highest MT expression at 5.93±0.25 μg MT mg protein^−1^, while other cell lines showed lower MT expression ranging from 1.54±0.06 to 3.21±0.19 μg MT mg protein^−1^. The ratios of induced MT/basal MT were calculated, and interestingly, MCF7 cells showed the highest induction of MT by 10.79±1.36-fold, while other cell lines showed induction by 1.40±0.10 to 3.65±0.30-fold. Treatment with 10 μM CdCl_2_ for 24 h strongly induced MT in MCF7 cells, which are p53+/ER+, compared to a smaller induction in other cell lines that are p53−/ER− or p53−/ER±.

### Localisation of MT after exposure to cadmium

**Figure 3:**
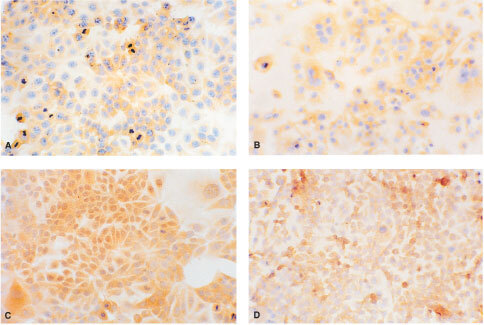
Immunohistochemical staining for MT with rabbit polyclonal anti-MT serum which cross-reacts with human MT. Control cells: MCF7 (**A**), and HCC1806 (**B**). Cells treated with 10 μM CdCl_2_ for 24 h: MCF7 (**C**), and HCC1806 (**D**). Positive staining is brown in colour. Magnification 400×.

The localisation of MT in MCF7 and HCC1806 cells after treatment with 10 μM CdCl_2_ for 24 h was determined by immunohistochemical staining using a polyclonal rabbit antibody to MT. Prior to CdCl_2_ treatment, only a light staining for MT was observed, mainly in the cytoplasm of both cell lines (Figure 3a,b

). After exposure to 10 μM CdCl_2_ for 24 h, intense staining was observed in the cytoplasm and nucleus of MCF7 cells (Figure 3c). In contrast, HCC1806 cells showed staining in the cytoplasm with a weak staining in the nucleus (Figure 3d). Negative control slides incubated with normal rabbit serum showed no staining for MT.

### Induction of MT mRNA expression by cadmium

**Figure 4:**
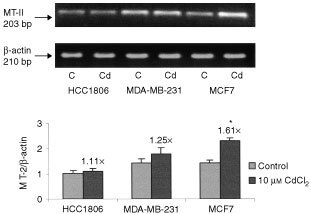
MT-II expression at 23 PCR cycles. RT–PCR was used to analyse total RNA samples derived from three epithelial breast cancer cell lines: HCC1806, MDA-MB-213, and MCF7. PCR products were electrophoresed on agarose gel and stained with ethidium bromide. Control cells are represented by C and cells treated with 10 μM CdCl_2_ for 24 h are represented by Cd. Intensities of cDNA bands were analysed by densitometry and MT-II mRNA expression was normalised with β-actin. Changes in MT-II mRNA expression following treatment were calculated as a ratio of treated cells/control cells and indicated in the graph. Results are mean±s.e. of three independent experiments. *Significantly different from control cells at *P*<0.05.

To investigate the induction of MT mRNA by cadmium, the cell lines MCF7, HCC1806 and MDA-MB-231 were treated with 10 μM CdCl_2_ for 12 h and mRNA levels were compared. Total RNA was isolated from the cell lines and 5 μg of RNA was subjected to RT–PCR analysis using a primer specific for human MT-II and a primer for β-actin simultaneously. PCR products were electrophoresed on agarose gel and stained with ethidium bromide. The intensities of the bands were measured and MT-II mRNA expression was normalised with β-actin. At 23 cycles of PCR, this analysis showed a significant increase in MT-II mRNA expression in MCF7 cells following CdCl_2_ treatment, but little change in HCC1806 and MDA-MB-231 cells was observed (Figure 4

). The induction of MT-II mRNA was calculated and expressed as a ratio of treated cells/control cells. Results showed a greater induction of MT-II mRNA expression in MCF7 cells (by 1.61±0.08-fold) than in HCC1806 cells and MDA-MB-231 cells (by 1.11±0.13 and 1.25±0.06-fold, respectively).

### Detection of apoptosis induced by cadmium

**Figure 5:**
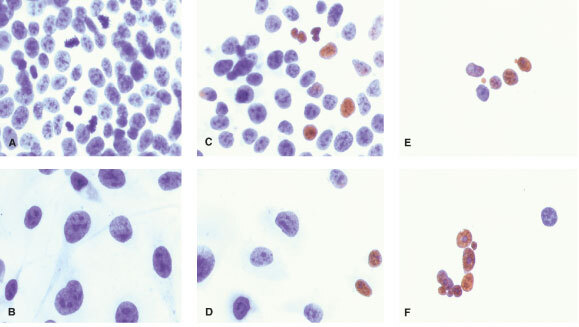
*In situ* detection of apoptotic bodies using TUNEL technique. Control cells: MCF7 (**A**), and MDA-MB-231 (**B**). Cells treated with 40 μM CdCl_2_ for 8 h: MCF7 (**C**), and MDA-MB-231 (**D**). Cells treated with 40 μM CdCl_2_ for 24 h: MCF7 (**E**), and MDA-MB-231 (**F**). TUNEL-positive apoptotic bodies are stained brown. Magnification 400×.

**Figure 6:**
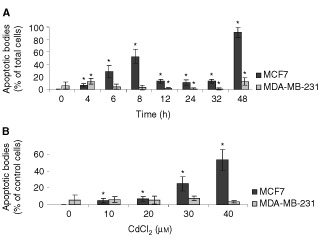
*In situ* detection of cadmium-induced apoptosis using the TUNEL technique. (**A**) MCF7 and MDA-MB-231 cells were treated with 40 μM CdCl_2_ for different time periods. (**B**) MCF7 and MDA-MB-231 cells were treated with various concentrations of CdCl_2_ for 8 h. Apoptotic bodies were counted in randomly selected fields. Results are mean±s.e. of 10 randomly selected fields. *Significantly different from control at *P*<0.05.

The terminal deoxyribonucleotidyl transferase mediated dUTP nick end labelling method (TUNEL) was used to determine the number of apoptotic bodies in epithelial breast cancer cells present following CdCl_2_ treatment. Based on the cell viability data, the concentrations of 10 to 40 μM of CdCl_2_ were selected to treat MCF7 and MDA-MB-231 cells for different time points between 4 to 48 h to induce apoptosis. Apoptotic cells were identified by the appearance of specific nuclear staining, condensed nucleus and/or formation of apoptotic bodies as examined at 400× magnification (Figure 5

). The number of apoptotic cells was expressed as a per cent of total cells counted. At 10, 20, 30 and 40 μM of CdCl_2_, the highest number of apoptotic bodies in MCF7 cells was observed between 6–8 h and again at 48 h (Figure 6a

, data shown only for 40 μM CdCl_2_ treatment). In MDA-MB-231 cells, apoptosis remained at a constant low level at all the time points examined between 4 to 48 h (Figure 6a, data shown only for 40 μM CdCl_2_ treatment). In MCF7 cells at all time points tested, the number of apoptotic cells increased as the concentration of CdCl_2_ was increased, while in MDA-MB-231 cells, there was again a constant low level of apoptosis (Figure 6b, data shown only for 8 h time period).

### Uptake of cadmium by different cell lines

**Figure 7:**
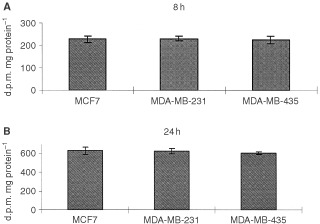
Uptake of CdCl_2_ by different cell lines. MCF7, MDA-MB-231 and MDA-MB-435 cells were treated with 10 μM of ^109^CdCl_2_ and disintegrations per minute (d.p.m.) were measured and expressed as d.p.m. mg protein^−1^ at (**A**) 8 h, and (**B**) 24 h after treatment. Results are mean±s.e. of three independent experiments.

The uptake of Cd by MCF7, MDA-MB-231, and MDA-MB-435 cells was determined by treating the cells with ^109^CdCl_2_ and then measuring radioactivity. The results are expressed as d.p.m. mg protein^−1^. The results show no significant differences in cadmium uptake in these cell lines at 8 or 24 h of treatment (Figure 7

). The data suggest a similar Cd uptake and uniform accumulation of Cd in these cell lines.

## DISCUSSION

This study demonstrates a potential role of p53 on the expression and induction of MT in human epithelial breast cancer cells. Our results show that epithelial breast cancer cells with differential p53 expression exhibit differences in induction of MT, and response to toxicity following treatment with cadmium. Although cadmium caused induction of MT in all cell lines regardless of ER and p53 status, MCF7 cells showed a much higher induction in MT protein and mRNA levels as compared to cell lines that are p53− and ER−/± (Figure 2). Cell viability results and TUNEL staining for apoptosis both showed a greater sensitivity of MCF7 cells to toxicity induced by cadmium as compared to MDA-MB-231 (p53−, ER−) and HCC1806 (p53−, ER−) cells (Figures 1 and 6). To investigate whether these differences are due to differential accumulation of cadmium, the uptake of cadmium in the cell lines was determined by treating the cells with^ 109^CdCl_2_ and calculating the radioactivity as ^109^Cd d.p.m. mg protein^−1^. Our studies on cadmium uptake showed that the different cell lines have similar cadmium uptake and accumulation (Figure 7). Thus, higher MT induction and greater sensitivity to cadmium-induced toxicity in MCF7 cells is not a consequence of greater accumulation of cadmium, but may be a consequence of positive p53 expression. The presence of p53 in MCF7 cells likely mediates the dose- and time-dependent apoptosis observed during treatment with CdCl_2_. In contrast, the absence of p53 in MDA-MB-231 cells appears to render the cells resistant to apoptosis when treated with CdCl_2_ (10–40 μM) from 4 to 48 h.

The presence of wild-type p53 and occurrence of apoptosis may be important indicators of MT expression, since the expression and induction of MT has been associated with protection against oxidative stress and apoptosis. Cadmium is a potent inducer of MT synthesis that not only acts through metal response elements of the MT gene to up-regulate gene transcription (Karin *et al*, 1984), but can also cause intracellular free radical generation and lipid peroxidation, DNA damage and a characteristic apoptotic response (El Azzouzi *et al*, 1994; Yang *et al*, 1997). Apoptosis induced by cadmium is preceded by the participation of oxidative stress, with up-regulation of oxidant stress genes such as glutathione S-transferase and γ-glutamylcysteine synthetase, activation of redox sensitive transcription factor AP-1 and NF-κB, as well as induction of MT-1 and MT-II synthesis (Hart *et al*, 1999; Zhou *et al*, 1999). Several mechanisms may be involved in the protective effect of MT against metal toxicity and apoptosis. MT has been shown to bind cadmium and to sequester it away from important cellular organelles (Goering and Klaassen, 1984). In addition, MT may have antioxidant properties because of its high cysteine content which may protect against DNA damage (Cai *et al*, 1996). A recent study has shown that treatment of MCF7 cells with 10 μM CdCl_2_ can increase MT-IIA isoform and can protect against cadmium toxicity (Méplan *et al*, 1999). Since MCF7 cells were much more sensitive to cadmium toxicity as compared to HCC1806 and MDA-MB-231, a higher induction of MT in MCF7 cells may be required to protect against cytotoxicity and DNA damage.

The tumour suppressor protein p53 plays a role in regulation of cell cycle progression, DNA repair and apoptosis. The p53 activity is sensitive to oxidative stress and exposure to heavy metals. These conditions and DNA strand breaks can induce p53 and initiate apoptosis in cells. In order to understand the role of MT in apoptosis and the effect of p53, we have used epithelial cells with p53+/ER+ along with epithelial cells with p53−/ER−, and the results suggest that only MCF7 cells with p53+ can induce MT and induce apoptosis. The p53 activity may influence the expression of MT by several potential mechanisms. The p53 protein, which is a transcription factor, can activate or repress the expression of genes in anti-proliferative pathways (Agarwal *et al*, 1998). It has been demonstrated that zinc binding is crucial for p53 protein stabilisation and DNA binding (Hainaut and Milner, 1993). Since MT may be involved in zinc homeostasis, it can regulate the supply of zinc to p53 protein for its optimum activity and also protect the cells from toxicity to metals and free radicals. Lastly, zinc is known to inhibit the activation of both caspase 3 (Perry *et al*, 1997) and Ca/Mg-dependent endonuclease (Cohen and Duke, 1984), which play critical roles in the execution of apoptosis. MT, as a zinc binding protein, may also act as an anti-apoptotic factor. Thus, the expression of MT and p53 may play a critical role to determine whether cells undergo apoptosis or proceed to cell cycle stages.

The localisation of MT has important implications on its functions. Positive staining for MT has been demonstrated in various human tumours and can be localised in the cytoplasm and nucleus, often depending on cellular differentiation and proliferation (Cherian and Apostolova, 2000). Both MCF7 and HCC1806 cells showed mainly cytoplasmic staining for MT prior to induction with cadmium (Figure 3a,b). Nuclear staining noted in a few cells were associated with proliferative activity as noted by morphology in the immunohistochemical staining. However, following treatment with 10 μM CdCl_2_, intense staining for MT was observed in the nucleus and cytoplasm of MCF7 cells, but not in HCC1806 cells (Figure 3c,d). The nuclear localisation of MT in MCF7 cells was associated with strong sensitivity towards cadmium-induced toxicity and the presence of p53. As mentioned, MT may participate in regulating p53 stability and DNA-binding activity, and in protecting cells against cadmium-induced toxicity, p53-induced reactive oxygen intermediates, and apoptotic DNA damage. These activities require MT to be present in the nucleus. This is in accordance with the suggested functions of nuclear MT: donation of zinc to transcription factors for DNA synthesis, regulation of gene expression, protection of the nucleus from oxidative damage, and protection of the DNA from damage and apoptosis (Cherian and Apostolova, 2000).

Activities of p53 are intricately linked to MT induction and localisation. p53 causes cell cycle arrest at G1 in response to low levels of stress (Schwartz and Rotter, 1998), and studies have shown localisation of MT in the nucleus occurs during G1/S phase when the requirement for zinc is highest (Cherian and Apostolova, 2000). p53 initiates apoptosis during high levels of stress (Schwartz and Rotter, 1998), and MT has been shown to play a role in different aspects of apoptosis. Further evidence is provided by a recent study showing that cadmium can affect the stability and DNA-binding activity of p53 (Méplan *et al*, 1999). In MCF7 cells, 10 μM of CdCl_2_ caused an increase in p53 protein level and DNA binding activity. However, at higher concentrations (20 μM or greater), cadmium down-regulated p53 protein levels and DNA-binding. Interestingly, in the present study, MCF7 cells showed a strong induction of MT only when treated with 10 μM CdCl_2_ and not higher concentrations (20–50 μM, data not shown). This demonstrates a correlation between strong MT induction and p53 activity.

Evidence points to a relationship between p53 and MT expression and induction in epithelial breast cancer cells. Higher basal MT expression is associated with p53 mutations in breast cancer cells (Friedline *et al*, 1998; Jin *et al*, 2000). Both factors indicate and may contribute to progression and poor prognosis of the cancer. In contrast, activation of p53 by cadmium is accompanied by high induction of MT. Besides protecting cells against cadmium-induced oxidative stress and DNA damage, MT may have an essential role in regulating p53 stabilisation and gene transcription, as well as protecting cells against p53-mediated apoptosis. Although the presence of ER may affect basal MT expression, the induction of MT seems to be unaffected by ER since cell lines weakly positive for ER did not show a significantly higher induction of MT compared to the cell lines negative for ER. Nonetheless, further studies are needed to ascertain the roles of p53 and ER in MT expression and induction. Additionally, more extensive work with different cell types may help to determine the tissue-specificity of any effect of p53 on MT induction.
